# Understanding weight management experiences from patient perspectives: qualitative exploration in general practice

**DOI:** 10.1186/s12875-023-01998-7

**Published:** 2023-02-13

**Authors:** Kimberley Norman, Lisette Burrows, Lynne Chepulis, Rawiri Keenan, Ross Lawrenson

**Affiliations:** University of Waikato, Gate 1, Knighton Road, Hillcrest, Waikato District Health Board, Pembroke Street, Private Bag, Hamilton, 3200 New Zealand

**Keywords:** Obesity, Primary care, Client view, Barriers, Effective weight management, New Zealand, Obesity healthcare

## Abstract

**Background:**

Obesity is a complex health issue affecting the quality of life of individuals and contributing to an unsustainable strain on healthcare professionals and national health systems. National policy guidelines indicate that general practice is best suited to deliver obesity healthcare, however, obesity rates continue to rise worldwide indicating interventions are ineffective in this space. The aim of this study was to explore the weight management experiences from patient perspectives.

**Methods:**

This qualitative study used semi-structured interviews with 16 rural Waikato general practice patients. Interviews were analysed using reflexive thematic analysis.

**Results:**

Four themes were identified: Inconsistent Information, Significance of Holistic Factors, Obesity Centre Need, and Education. Participants expressed frustration at contradictory health messages, commercial company and ‘expert’ definition distrust, and that ‘holistic’ aspects to health significant to the weight management journey were unable to be addressed in general practice.

**Conclusion:**

Whilst primary care is positioned as suitable for delivering obesity healthcare, this study found that participants do not perceive general practice to be equipped to deliver this care. Instead, participants argued for a specialist obesity centre capable of meeting all their obesity healthcare needs. Further, wider issues including on-line commodification of health and neo-liberal capitalism - factors that exploit people with a stigmatised health issue - can cause further harm to the participant. A radical modernisation of education, information, and resources from regulated, qualified and ‘trusted’ healthcare professionals who can provide safe, non-stigmatising supportive services is recommended to meet the unique and changing food climate, reduce obesity rates and improve health outcomes.

## Background

Obesity affects over 650 million people across the world [1] and leads to further physical, psychosocial and cultural health issues [2, 3]. Obesity and its related comorbidities reportedly cost over US$990bn globally [4], which is unsustainable and threatens to bankrupt national health systems, including the UK National Health Service (NHS) [5]. The predominant clinical view of obesity is that it presents a significant health risk. However, many other perspectives of obesity exist, including obesity not being classified as a health risk as well as obesity being the preferred ‘ideal’ in some cultures [6–8] and therefore not warranting clinical ‘intervention’ or ‘treatment’. However, obesity is a stigmatized health concern in western contexts with discrimination reportedly experienced in all levels of life [9–13] further contributing to a reduced quality of life [4].

The World Health Organisation defines obesity as preventable and reversable through effective weight management strategies [14]. The national health systems in the UK, Canada, Australia, America and New Zealand (NZ) [15–19] position primary care as suitable for obesity healthcare. Guidelines recommend routine identification and treatment of obesity in primary care to reduce obesity rates [16, 20]. The Body Mass Index (BMI) is (while arguably a flawed tool [21] used to measure obesity levels in primary care, with a healthy weight range classed as 18.5-24.9, overweight between 25 and 29.9, and obese over 30 [22]. However, BMI is reported to be under-recorded and weight loss interventions under-referred [23] in general practice. Weight management options are available through general practice, privately, through commercial avenues, or internet-based sources [19, 24, 25]. In NZ, primary care offers weight management advice via national guidelines [19]. Secondary care referral options for a clinician include dietitian consultations, weight management medication, hospital weight management clinic and bariatric surgery [19]. In so saying, there are limited publicly funded spaces in these programmes, and options such as bariatric surgery, low calorie diet plans or exercise establishment memberships, are increasingly being offered to patients who can self-fund or pay for private health insurance [26, 27]. However, many people at risk of developing obesity live in high-deprivation and are financially unable to access this care [28], which can contribute to increasing the health inequity gap [29].

Achieving weight management has been argued to be simply an issue of balancing an ‘energy in versus energy out’ eq. [30]. However, evidence indicates it is more complex, due to a myriad of additional contributing factors, including the obesogenic environment, psychological factors, sociocultural norms, adverse or traumatic life events, colonisation impacts (for indigenous populations) and social determinants of health [2, 6, 31–34]. While acknowledging the significant role modern obesogenic environments and an individual’s choice to engage with weight management plays, one of the most effective ways to achieve weight management is through a combination of diet, exercise, and behavioural change conducted in culturally appropriate ways [19, 35]. This combination and balance of factors needs to be calibrated to the individual for suitability as no one diet suits all. Despite this literature, obesity rates continue to rise worldwide, including the UK, suggesting there are barriers as current weight management interventions in general practice are ineffective.

NZ has high obesity rates with 34% of adults classed as obese [28]. There is significant health inequity experienced by the Indigenous Māori population in NZ with 51% obesity rate, as well as Pacific Island populations in NZ at 71% [28]. While effective management of weight is complicated and influenced by many compounding factors both within and outside the scope of general practice, Māori also face additional challenges when engagaing with public health systems such as experiences of hostility, alienation, racism and trying to navigate a health system that does not align with a Maori health worldview reported [36]. Yet, there is limited literature that focus on the experiences of weight management in general practice from the patient perspective [37–41]. NZ populations at high risk of developing obesity include rural communities, Pacific and Indigenous Māori populations, and those living in high-deprivation areas who experience inequities [28, 42]. The aim of this study was to explore the patient perspectives of their weight management experiences in general practice to identify barriers and ways to improve health outcomes. It is hoped that this study will suggest new ways to offer weight management strategies within general practice and the community.

## Method

### Participants

Participants were recruited through rural general practices.. Participant criteria included: aged > 25 years, currently or recently resided in a rural Waikato location, not on any weight influencing medication, and identified as having had some experience with weight management in general practice context. While acknowledging the subjective nature of obesity and the definition of ‘obesity’ being socio-culturally influenced [43–45], for the purposes of this research the clinical measurement of obesity was used to demarcate weight and identify participants who were eligible for this study (BMI of > 30) [46].

### Data collection

Rural general practices and Māori healthcare providers across the Waikato region were contacted via phone and email and invited to participate. They were asked to identify and pass on the female researchers (KN) details (or gain consent to be contacted by the researcher) to any of their patients they saw that fit the criteria for this study. Seven participants were recruited through this avenue. Due to the potential that some patients who fit the criteria of the study may not have visited their general practice recently, a snowballing strategy [46] was utilised. All recruited participants were invited to share the researchers details to those in their community they knew who might want to take part. Ten participants were recruited from this method across the Waikato region. Purposeful sampling was conducted towards the end to recruit males, as only one out of the first 14 participants was male. Three males were recruited, however one male participant was excluded during the interview weight gain was found to be influenced by medication which was an exclusion criterion. A total of 16 participants were recruited and the demographic details are in Table 1.

**Table 1:** Participant demographic data

Demographic	Participants (n)
Male	3
Female	13
Māori / NZ European and Māori	8
Non-Māori	8
Age 25- 45 years	8
Age 46-70 years	8
High Deprivation Residence [47]	16

Information sheets and consent forms were given to all participants, rapport was built with the participants, the reasons behind why the study was being conducted, as well as any questions or concerns answered before consent was signed and interviews commenced. Interviews were held in person or via Skype at a time and place preferred by the participant [48]. A Māori cultural advisor and GP was included and contributed by guiding the research process, including processes such as karakia (Māori prayer), whakawhanaungatanga (process of establishing relationships), koha use (gift and gratitude for participant), and contributing to interpretation of Māori narratives in a western health context. While the interviewer identifies as non-indigenous, she has lived experience with significant weight management, and extensive experience in qualitative interviews and analysis, including awareness of the limitations of her own experiences when collecting or interpreting indigenous narratives and actively sought guidance throughout the study. These factors contributed to reducing power imbalances. No participants wanted copies of transcripts. Ethical approval was granted by the University of Waikato Human Research Ethics Committee reference HREC2020#38.

Interviews were semi-structured and guided by a set of questions to ensure that all participants were asked the same open-ended / exploratory questions, and to ensure participants had agency to share their story in their own words. The open-ended interview questions included: ‘could you please tell me about your experience with weight management?’ and ‘could you please tell me about your experience with any barriers to weight related health engagements?’ All participants were encouraged to speak about their experience for as long as they wanted to. Interviews were audio recorded for later transcription, notes were taken by interviewer, participants were thanked and compensated for their time with a $30 voucher.

### Analysis

All interview data was transcribed verbatim and analysed using reflexive thematic analysis [49]. Each transcript was printed out, read and re-read by the researcher for immersion in the data. In the right-hand margin of each transcript, sections of conversation were analysed and labelled with no pre-defined categories, enabling the concepts that were significant to the participants’ experiences to be identified and highlighted. These ideas and interpretive notes were used for the codes of this study and were checked by second researcher. Each transcript was analysed in turn, and then re-analysed for any missing codes. The Māori cultural advisor read and ensured that appropriate interpretation occurred for all participants identifying as Māori. Once all codes were listed, any redundant or double-up codes were removed. KN, RK, and LB were all involved with analysis and formulation of themes. Whilst Braun and Clarke highlight that the ability to achieve data saturation is situated and subjective [50], this analysis found no new themes when revisiting the transcripts and reflecting on codes already identified.

## Results

All interviews lasted up to 60 minutes. Six initial themes were formed from the coding lists and after reflection of the transcripts, four overarching themes were identified: Inconsistent Information, Significance of Holistic Factors, Obesity Centre Need, and Education.

### Inconsistent information

Inconsistent information around food dietary advice was expressed as significant in the weight management process. One woman reported “*Knowing how many calories to eat is what I struggle with”* (Participant 04). Despite accessing multiple health ‘sources’ and ‘professionals’, the actual calorie deficit amount for her weight management journey was still a mystery – making her weight goals unachievable before even starting her diet plan:

*“What actually is it? You put it in my fitness pal [app], it’s 2500 [calories]. You do a [gym] body scan, it’s 2700. I went to [commercial nutritionist] it was 2200. There’s like 200-300-500 calorie difference- it’s a whole meal!”* (Participant 04)

Popular diets such as Ketogenic (low carbohydrate diet [19]) provoked tension for some. One participant described concern about going on a Keto diet saying that it is *“actually bad for you”* (Participant 09) after being advised to try it. Another participant declared that the concept of only eating fats to lose fat went against his ‘general’ understanding of weight loss whereby “*It’s kind of like the opposite of everything you learn of good nutrition”* (Participant 17).

Accessing quality and reputable information became a “*struggle”* (Participant 04) as participants described many self-advertised weight related health ‘professionals’ (outside general practice and commonly in the commercial sector) as unqualified to give accurate dietary advice. Participants expressed that the ability to rely on dietary information became unachievable as there was “*so much misinformation available to everyone”* (Participant 09). Confusion around what to believe caused further tension:

*“There is contradictory information out there”* (Participant 09)

The consistent misleading or confusing information was expressed with feelings of helplessness and powerlessness to achieve their weight goals. As summarised by one participant:

*“Where- what do you trust?”* (Participant 09)

Visiting their general practice for dietary advice was not actioned by all participants. Some participants highlighted that they did not think their GP would be a place for this type of health advice:

*“Going to the GP would be like a last resort”* (Participant 11)

And sometimes actively avoided:

*“I don’t think I’ve ever gone to a GP [solely for weight management advice]- but I don’t think I would, because I don’t think it would benefit me. My perspective of it is I feel like all they would say is ‘eat better and go to the gym’ And that’s what I’ve been currently trying to do”* (Participant 04)

Experiences with weight management options through a GP varied. Medication was *“extremely expensive”*, made one participant *“violently ill”* (Participant 14) and others had heard “*traumatizing things about the side effects”* (Participant 11) of particular medication. GPs were approached for bariatric surgery as one participant described, “*I had to be GP referred to go privately”* (Participant 14)*.*

Commercial weight management programmes were viewed with scepticism as one woman reported:

*“There is all these different companies that are just trying to make money and like, [commercial business] they’re all businesses, they’re all trying to make money. Like, yes they are trying to ‘help’ people, but they’re also a business that’s trying to make money*” (Participant 11)

Advertising of weight management through ‘X week challenges’ from commercial gyms implied ‘expertise’. As one participant highlighted this presumed ‘expertise’ ended up being generalised nutritional advice and she got “*really nothing out of it”* (Participant 04). Her failure to reap any rewards from this advice generated further disappointment, frustration and depression:

*“How is it that I followed the nutrition plan and worked out for like four or five days a week and I lost 800 grams?! I was just so heartbroken. I was like -what’s the point? I’m trying so hard and it’s just not working. So then I could that kind of sent me back on a downward spiral”* (Participant 04)

Further confusion and tension surrounded the definition of a business operating as a weight management ‘expert’ as there was little transparency in terms of qualifications. Trying to identify who was a reliable information resource among all the available sources was difficult for many. One participant highlighted their frustration at wanting to find a reliable weight management professional:

*“I said to [doctor] I’ve been to a nutritionist, and it didn’t really fit me what that nutritionist has given me. I don’t know enough information about a difference between a nutritionist and a dietitian, do you think it would be better for me to go to a dietitian, like I’m happy to pay to go I just don’t know the differences easily. Or do I try a different nutritionist? Like, I want to get my food right!”* (Participant 04)

### Significance of holistic factors

For those who had achieved their weight loss goals, or who had achieved some weight loss in the past, a healthy mind set was crucial. Prior to losing weight, understanding why she ate was important for her success and adherence to her choice of calorie deficit plan:

*“I had to learn the association of what I did when I was depressed or feeling down, you know, I ate.”* (Participant 01)

Recognising personal relationships with food and eating behaviour were vital for any dietary changes to take place. Emotional connections to food, emotional eating, or using food to feel ‘good’ were identified as reasons for weight gain in some participants’ journeys:

*“I have changed my entire mental health, mental shift and food association with mental health so I don’t need chocolate to make me feel good anymore.”* (Participant 01)

*“I think people’s mental health has a direct impact on weight loss. And when you’re depressed, you just eat crap. You eat crap, because you feel like crap and you think you’re crap”* (Participant 14)

Psychological aspects to weight management were also recognised as contributors to eating behaviour:

*“Part of the problem for me is my depression and anxiety. When they play up I tend not to pay as much attention to what I’m eating and not eating and things like that”* (Participant 09)

Participants reported the need for a ‘holistic’ view of weight management that incorporated many aspects to weight management and “*not just my diet*” (Participant 08) as it would “*just be a better way”* (Participant 09)*.* One participant indicated:

*“[I need to] have my complete entire well being checked out- my mind, my spirituality, my environment”* (Participant 08)

Whilst another highlighted that balancing both physical and holistic aspects to weight management was key for effectiveness:

*“[It’s] very holistic, but also very scientific. This is why you do what you do. And this is why your body is reacting the way it’s reacting”* (Participant 14)

Feelings of failure were significant to further psychological harm with one participant reporting the whole experience being “*really disheartening”* (Participant 02). Another participant described being *“stuck in a cycle”* (Participant 04) of failed diets and that:

*“It makes me feel like shit, to be fair, because I feel like I’m doing something wrong”* (Participant 04)

### Obesity health centre

Participants expressed a desire for a service that could meet their weight management needs. The concept of a “*health centre rather than a medical centre”* (Participant 07) or “*weight care centres”* (Participant 06) was reported as a desired ‘place to go’ for these participants for weight management needs.

Weight management centres were positioned as a service that could provide reputable and reliable information as well as access to qualified health professionals who could help these participants. One woman described that having *“more access to information”* (Participant 14) was crucial, while another participant highlighted:

*“There needs to be somewhere where there is clear information from the government or actually from the medical professionals, saying, ‘This is what you can do to be better’”* (Participant 09)

Participants reported a significant desire and expectation that health professionals are proficient in the complexities of weight management:

*“Someone who is qualified and done research and knows what they’re talking about, and had experience with this, people, situations, so they know not every [diet] works for the same people”* (Participant 04)

*“I want to be able to have access to a practitioner that understands the multi-dimensional layers to obesity”* (Participant 08)

Expectations on a single health professional to provide all the needs for weight management were low due to the variances of weight management needs. Difficulties with trying to deal with a health issue that is “*not just black and white”* (Participant 02) with a GP only having *“10 minutes to make that assessment”* (Participant 16) was highlighted as an issue that needs addressing. One participant expressed:

*“It’s probably really hard to find someone like that [to cover complexities]. But if one person can’t do it, get a team, you know?”* (Participant 02)

### Education

Whether participants had achieved their desired weight, or were still on the weight management journey, all participants positioned education about healthy living as important.

The change in societal norms was described in many forms. One participant highlighted disgust that advertising and processed foods companies are using discourse such as *“organic sweetening agent e105a”* as a way of *“hiding what [sugar level] is in”* (Participant 03) their food products.

Education around processed food labels was positioned as vital to one participant’s success at weight management:

*“Anything with a square on it explaining what’s in it, to me that’s a warning sign”* (Participant 03)

Education in schools was positioned as crucial to save the next generation from suffering from obesity. Teaching them how to cook food that “*could actually fuel you and taste good”* (Participant 14) and the need for teaching to be about *“healthy kai (food)”* (Participant 06) was important. As one participant expressed, the youth are *“the victim of the sugar”* (Participant 03).

Awareness that the weight management “*wasn’t a diet- it was a lifestyle”* (Participant 01) was crucial for long-term effectiveness. As one participant indicated:

*“Teaching about healthy food choices in teaching about healthy, what healthy bodies actually are is important”* (Participant 09)

## Discussion

### Summary

This study demonstrated many aspects to the patient experience of weight management including not only the need for a suitable calorie deficit dietary plan, but also addressing holistic aspects to their health such as psychological or cultural related experiences with weight. Expressions of confusion, frustration and deception around weight management advice and commercial sources of ‘help’ were found to be pervasive. Patients reported wanting education from ‘trust-worthy’ qualified professionals who could meet their wider health needs, a feat in which a GP could not achieve in their small 10-minute consultation. Surprisingly, minimal discourse linked weight management to general practice or interventions and some explicitly highlighted they would not consider visiting their GP for weight advice.

### Strengths and limitations

As with any qualitative study, the unconscious bias from researchers can influence design and analysis. Recognising the potential for bias, this study was designed and analysed by a team of academic, general practitioner, and lived obesity experience researchers which actively included processes of cultural awareness and reflexivity throughout the research entirety. While qualitative findings cannot be generalised, this research provides novel insights to the experience of weight management from the patient perspective, which is imperative to understand if any future weight management interventions are to be effective. While the sample size was small and rurally based, it is relevant to all people attempting weight management. The research achieved saturation in the interviews with themes consistent across narratives and no new themes emerging. However, it is acknowledged that whilst the experiences and themes from both Māori and non-Māori participants were similar throughout this study, using an indigenous health worldview lens would likely elicit a wider range of themes and understandings for Māori participants..

### Comparison with existing literature

An unexpected finding was the lack of discourse around weight management experiences in general practice, despite this being the context for this research. Many patients positioned general practice as unsuitable to deliver effective weight management healthcare, a perspective that contradicts the national health policy and clinical guidelines in the UK, America, Australia, Canada and NZ [15–19]. When general practice was talked about, it was positioned with negative clinical options (such as medication and bariatric surgery), and an overall inability to provide the obesity management patients desired. For example, addressing the holistic needs, including spiritual and cultural factors, to weight management and lifestyle habits was positioned as unsuitable for the time-poor consultation with a GP. Further, some patients specifically stated they would not even engage with their GP for weight management as it was viewed as ‘unhelpful’, which supports one UK study where patients did not see the GP or NHS as appropriate for this healthcare [51]. It is little wonder that obesity and obesity comorbidity rates are increasing in the UK, and worldwide, given that not only do GPs experience many barriers to effective obesity healthcare delivery in their practice [52–54] but their patients potentially do not come to them for this healthcare in the first place.

Instead, many patients who chose to engage with weight management did so through non-general practice avenues such as fad diets or commercial companies. However, significant dis-trust, confusion, and feelings of deception were associated with these options. Commercial companies selling ‘personalised’ programmes for weight loss results that premised on very little ‘science’ were commonly reported throughout these narratives. With obesity stigma and the ‘thin ideal’ (a body image concept that is promoted to be aspired to) being pervasive in Western culture [6, 12, 13, 55] it is unsurprising that commercial endeavours such as private companies and food marketing tactics [56] would be used to exploit those who are ostracized and vulnerable. One UK study [51] explored patients experiences of a GP (and therefore ‘reliable’) referral to commercial weight loss programmes was welcomed as patients viewed weight as more lifestyle issues requiring a non-medical solution. However, participants in this study highlighted that their commercial weight management programmes failed to meet their comprehensive needs, and only addressed one layer of the complex weight management experience (either food, exercise or behaviour change) which contradicts the national guidelines and effective weight management literature in the UK and NZ [2, 16, 19].

Issues around neo-liberal capitalist behaviours were also noted by participants whereby products consistently acted in ways that ‘hide’ sugar content and using language to imply they are qualified to give specialist advice (nutritionist versus dietitian for example), further deceiving the individual seeking help. Whilst some participants understood the economical concepts of weight loss programmes, the ‘service’ or ‘product’ they paid for did not meet their expectations despite being advertised as ‘effective’. This generated more confusion about where to go for help, what to ‘believe’ anymore or who the ‘experts’ actually are for all patients. Clear information about nutrition and exercise was desired by these patients supporting previous findings [37, 57]. However, this study found the information or ‘education’ sought after transcended the ‘reductionist’ nutritional or lifestyle weight management advice of previous findings [19, 30, 41] and included factors such as how to navigate this current obesogenic climate and avoid consumer ‘traps’.

Surprisingly, patients called for the establishment of an obesity healthcare centre. This ‘one-stop obesity shop’ was positioned to provide holistic obesity services that could extend beyond a GP (in)capability and not have a financial interest in repeat business that commercial avenues were viewed to have. Facilitating access or providing care for the myriad of factors that are recognised to contribute to obesity including culturally appropriate services for indigenous populations was stressed as crucial for successful weight management. Previous literature has also indicated that trauma and adverse life events can contribute to weight [34], indicating that obesity healthcare could benefit from including psychological services such as counselling as a way to improve some patients’ relationships with food and extend beyond programmes that only include dietary manipulation and exercise increase. In addition, this centre could mitigate the confusion and ‘dis-trust’ experienced by patients through employing regulated health professionals, or ‘actual experts’ that could offer reliable ‘trust-worthy’ weight advice. While the capacity for general practice to provide obesity healthcare has been questioned in previous urban literature with many barriers identified [44–46, 51–53], this study extends this need for a specialised obesity referral service to indigenous and rural areas who experience significant health inequities.

### Implications for clinical practice

This study found the patients perspective did not fully align with the national position that general practice is ‘best suited’ for effective obesity healthcare. Future research should investigate the percentage of patients utilising general practice for weight management as these efforts could be mis-placed. Further, an appraisal focused on the suitability of general practice to provide obesity healthcare is strongly recommended, as this was found to be questionable and potentially, hindering obesity reduction efforts before attempts are even made.

In addition, research into the feasibility of an obesity centre establishment is recommended as this could reduce the strain on general practice and provide patients with comprehensive, culturally appropriate healthcare. Many participants felt that their ‘holistic’ obesity related health needs were not met in their general practice and desired access to a helpful referral pathway which was positioned as a ‘trustworthy’ source of information through their primary care clinician. Potentially, an effective primary care health service for obesity could be one that supports a specialised secondary service that can meet the ‘holistic’ health needs of patients. Previous literature has indicated that primary care is a valuable system that can contribute to better health outcomes and equity [58]. Investigation into the development of obesity health services and how the division of work between primary and secondary care should be explored for efficacy purposes in the future.

Public health education on obesity management urgently needs updating to include wider aspects to weight management besides calorie manipulation. Education needs to include factors within the reach of the individual, such as the ability to comprehend food labels, understanding biomedical responses to lifestyle factors, cultural influences on food consumption, and an awareness of personal psychosocial behavioural connections with food. However, the wider political climate also needs to be understood, regulated and held accountable for the factors that directly influence the individual’s ability to engage with a healthy lifestyle.

## Data Availability

The datasets generated and analysed during the current study are not publicly available due to the small rural geographical location where data was collected and the potential for identifying participants. The datasets are available from the corresponding author on reasonable request.

## Abbreviations

UK: United Kingdom
NZ: New Zealand
BMI: Body Mass Index
GP: General Practitioner
